# Exploring the Efficacy of Conservative Management Versus Surgical Intervention in Anterior Cruciate Ligament (ACL) Injuries: Insights Into Reinjury Rates, Quality of Life, and Long-Term Outcomes

**DOI:** 10.7759/cureus.74718

**Published:** 2024-11-29

**Authors:** Daniel E Onobun, Chijioke Orji, Ethel O Ojo, Ajibola A Adebisi, Kenechukwu Igbokwe, Rajinder Sehmar, Reginald Ononye, Olgun Aksaray, Stephen Oputa, Chidera S Nmereole

**Affiliations:** 1 Orthopedics and Trauma, Warwick Hospital, South Warwickshire University NHS Foundation Trust, Warwick, GBR; 2 Orthopedics, Liverpool University Hospitals Foundation Trust, Liverpool, GBR; 3 General Surgery, Epsom and St. Helier University NHS Foundation Trust, London, GBR; 4 Trauma and Orthopedics, Gateshead Health NHS Foundation Trust, Gateshead, GBR; 5 Urological Surgery, Stepping Hill Hospital, Stockport NHS Foundation Trust, Stockport, GBR; 6 General Medicine, Altnagelvin Area Hospital, Londonderry, GBR; 7 General Surgery, University of Benin, Benin, NGA; 8 Business Administration, Kaplan Business School, Sydney, AUS

**Keywords:** acl injuries, conservative management, long-term outcomes, meta-analysis, quality of life, reinjury rates, return-to-sport, surgical intervention

## Abstract

This systematic review and meta-analysis evaluate the comparative outcomes of conservative management versus surgical intervention for anterior cruciate ligament (ACL) injuries. A comprehensive search of PubMed, Cochrane Library, Web of Science, SPORTDiscus, and Google Scholar focused on studies published between 2010 and 2024. Data synthesis employed both thematic analysis for qualitative data and meta-analysis for quantitative outcomes.

Key findings indicate that conservative management yields lower reinjury rates and higher quality of life (QoL) scores (64.6 vs. 57.1) compared to surgical intervention, though differences were not statistically significant (p = 0.1483). Both strategies demonstrated comparable return-to-sport (RTS) rates. Meta-regression analysis revealed that longer follow-up durations positively influenced outcomes (p = 0.0288). The average complication rate was 49.83 per study (CI: 25.84-73.82), underscoring significant risks associated with both approaches.

Conservative management shows promise as an initial treatment option for certain ACL injuries, particularly regarding reinjury prevention and QoL improvements. However, surgical intervention remains crucial for specific cases requiring enhanced knee stability. The results underscore the importance of individualized treatment planning, long-term follow-up, and further standardization of outcome measures in future research.

## Introduction and background

Anterior cruciate ligament (ACL) injuries are a common and debilitating condition, particularly among individuals engaged in athletic activities [1]. Despite advances in treatment, they remain a significant challenge due to their implications for knee stability, functionality, and long-term health. These injuries are typically managed through conservative approaches or surgical interventions, with the choice of treatment generating considerable debate among healthcare providers [2]. Conservative management focuses on non-invasive strategies, such as physiotherapy, resistance exercises, and modifications to physical activity, aiming to harness the body’s natural healing mechanisms to restore stability and function [3,4]. In contrast, surgical intervention, commonly involving ACL reconstruction, uses grafts to restore the ligament and knee stability [1]. The decision between these approaches is influenced by factors such as patient age, activity level, associated injuries, and preferences, as well as broader implications for healthcare outcomes and costs [5,6].

The long-term consequences of ACL injuries, including the risk of reinjury and progression to osteoarthritis, remain a critical area of investigation [7]. Studies have highlighted both the benefits and limitations of surgical and conservative treatments. Hohmann et al. reported that surgical reconstruction improved patient-reported outcomes and knee stability but found no significant advantages in activities of daily living or osteoarthritis prevention [8]. Migliorini et al. found that ACL reconstruction restored joint laxity more effectively than conservative management but was associated with a higher risk of osteoarthritis, raising concerns about long-term joint health [7]. Further studies, such as those by Shukla et al., suggested that while surgical intervention may initially yield better functional outcomes, these benefits diminish over time, with no significant differences between surgical and conservative approaches in the long-term risk of osteoarthritis [9,10]. Additionally, Hinterwimmer et al. noted the limited quality of randomized trials supporting universal surgical treatment, emphasizing the need for robust, long-term studies to guide clinical decision-making [11].

The ongoing debate underscores the need for a comprehensive meta-analysis to consolidate existing evidence and provide clearer guidance. Such an analysis is critical to addressing inconsistencies in the literature and determining the relative merits of each treatment. Moreover, the long-term implications of ACL management, particularly concerning osteoarthritis risk, remain a significant knowledge gap that a systematic review could address. Understanding how treatment outcomes vary across patient subgroups, including differences in age, activity levels, and associated injuries, is essential for tailoring treatment to individual needs.

The objectives of this meta-analysis are to compare the complications, functional outcomes, and influencing factors of conservative management versus surgical intervention in ACL injuries. By synthesizing diverse findings, the study aims to clarify the risks and benefits of each approach, provide insights into long-term outcomes such as quality of life (QoL) and reinjury rates, and identify patient-specific factors that influence treatment effectiveness. This evidence is expected to improve clinical guidelines and decision-making, ultimately enhancing patient care and outcomes in ACL injury management.

## Review

Methodology

Study Design

This systematic review and meta-analysis aimed to evaluate the comparative effectiveness of conservative management and surgical intervention for ACL injuries. The study adhered to the Preferred Reporting Items for Systematic Reviews and Meta-Analyses (PRISMA) guidelines to ensure transparency, accuracy, and reproducibility throughout the review process.

Search Strategy

A comprehensive search of the literature was performed to identify relevant studies published between 2013 and 2023. Five electronic databases - PubMed, Google Scholar, Cochrane Library, Web of Science, and SPORTDiscus - were systematically searched. The search strategy was designed to identify studies comparing conservative and surgical treatment approaches for ACL injuries. Boolean operators “AND,” “OR,” and “NOT” were employed to combine search terms such as “anterior cruciate ligament,” “ACL injury,” “conservative management,” “non-surgical treatment,” “surgical intervention,” and “ACL reconstruction.” Both Medical Subject Headings (MeSH) terms and free-text keywords were utilized to optimize search results.

To ensure inclusiveness, the search strategy was tailored to each database. Additionally, hand-searching of reference lists from included studies and relevant systematic reviews was conducted to capture any potentially missed studies. Only English-language publications were included to maintain consistency and accessibility of data.

Inclusion and Exclusion Criteria

Eligibility criteria were meticulously defined before the review process to ensure consistency and relevance. Studies were included if they focused on adult patients aged 18 years or older with primary ACL injuries. To meet inclusion criteria, studies had to compare conservative management with surgical intervention and report specific outcomes, such as knee function, return-to-sport (RTS) rates, re-injury rates, or the development of osteoarthritis. Additionally, eligible studies required a minimum follow-up period of two years to ensure the evaluation of long-term outcomes. Only studies published in peer-reviewed journals using randomized controlled trials, cohort studies, or retrospective designs were considered.

Exclusion criteria ruled out studies involving pediatric populations or patients with skeletal immaturity. Studies exclusively addressing revision ACL surgeries or multi-ligament injuries were also excluded. Further exclusions included studies with follow-up durations of less than two years, those lacking sufficient data for effect size calculation, conference abstracts, grey literature, and non-peer-reviewed articles. Additionally, studies published in languages other than English or before 2013 were not considered

Study Selection Process

The study selection process followed PRISMA guidelines. Initially, the titles and abstracts of all identified studies were screened to assess their relevance based on the inclusion and exclusion criteria. Full-text articles of potentially eligible studies were then retrieved for detailed evaluation. Duplicate studies were removed using Mendeley reference management software, which also facilitated the organization and management of retrieved references.

Three independent reviewers assessed the eligibility of each study. Discrepancies were resolved through discussion, and if necessary, a fourth reviewer was consulted. The PRISMA flowchart illustrates the selection process, including the number of studies identified, screened, assessed, and ultimately included in the final analysis.

Data Analysis

A meta-analysis was performed to calculate pooled estimates for key outcomes, such as knee function, RTS rates, and re-injury rates. A random-effects model was applied to account for anticipated heterogeneity among the studies. Heterogeneity was assessed using the I² statistic, with values above 50% indicating substantial variability. Publication bias was evaluated using funnel plots and Egger's test. When publication bias was detected, the trim-and-fill method was applied to adjust for potential distortions.

Pooled estimates for outcomes were calculated as odds ratios (ORs) with 95% confidence intervals (CIs). Forest plots were generated to visually represent the results, with individual study effect sizes depicted as squares and the overall effect size represented by a diamond. Sensitivity analyses were performed by excluding studies with a high risk of bias to assess the robustness of the findings.

Results

A total of 357 studies were identified through the initial search of databases, including PubMed, Google Scholar, Cochrane Library, Web of Science, and SPORTDiscus, as well as reference screening. After removing 114 duplicates and studies that did not meet the inclusion criteria, 243 studies were screened based on their titles and abstracts. Of these, 219 studies were excluded for not meeting the eligibility criteria. Full-text reviews were conducted on 24 studies, and only 20 were able to be retrieved. Six further were excluded and ultimately, 13 studies were included in the systematic review and meta-analysis.

**Figure 1:**
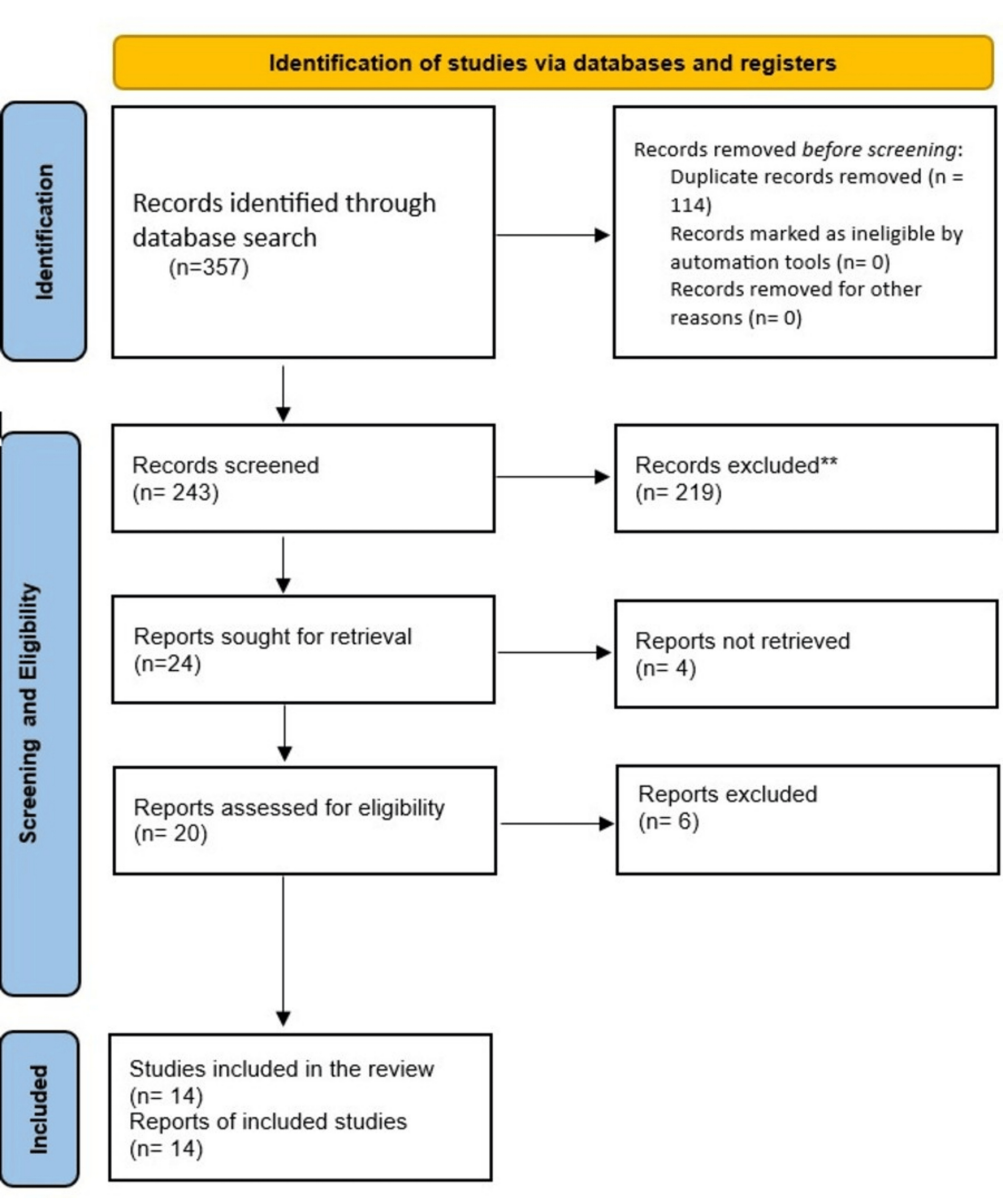
PRISMA flowchart

The PRISMA flow diagram in Figure 1 illustrates the study selection process, detailing the number of studies excluded at each stage. The data extraction table (Table 1) details the information and findings of these papers.

**Table 1:** Data extraction table

S/No	Articles/year	Main objectives	Methods used	Findings/conclusion
1	Grindem et al. (2014) [12]	The study objective was to compare outcomes between surgical and nonsurgical treatment of ACL injuries.	Prospective cohort study	There were few differences between the clinical courses following nonsurgical and surgical treatment of ACL injury in this prospective cohort study.
2	Sun et al. (2015) [13]	The study compared acute and chronic anterior cruciate ligament (ACL) reconstruction using ligament advanced reinforcement system (LARS) artificial ligament in young active adults with a 5-year follow-up.	A prospective randomized study	These results suggest that earlier ACL reconstruction using a LARS artificial ligament may provide an advantage in the treatment and rehabilitation of ACL rupture.
3	Harris et al. (2015) [14]	To compare, in young active adults with an acute anterior cruciate ligament (ACL) tear, the mid-term (five-year) patient-reported, and radiographic outcomes between those treated with rehabilitation plus early ACL reconstruction and those treated with rehabilitation and optional delayed ACL reconstruction.	Extended follow-up of a prospective randomized controlled trial.	At five years, patients assigned to rehabilitation plus early ACL reconstruction did not differ significantly in patient-reported or radiographic outcomes from those assigned to initial rehabilitation with the option of having a later reconstruction if needed.
4	Tsoukas et al. (2016) [15]	This study aimed to record and compare the functional and activity levels as well as the manifestations of osteoarthritis after isolated ACL ruptures between patients with conservative treatment and ACL reconstruction with hamstring tendon graft.	A randomized controlled trial study	ACL reconstruction using hamstring autograft resulted in better functional outcomes and laxity measurements than ACL-conservative management.
5	Dawson et al. (2016) [16]	This study aimed to assess the short-to-medium-term results of patients who chose conservative management in comparison to patients who had reconstructive surgery within the same period.	Retrospective study of 63 patients with ACL injury.	There were no significant differences in outcomes between ACL reconstruction and conservative management. - Patients who chose conservative management achieved similar function and satisfaction as those who had ACL reconstruction. - There is currently no clear evidence that one treatment approach is superior to the other.
6	Monk et al. (2016) [2]	To assess the effects of surgical versus conservative interventions for treating ACL injuries.	A randomised controlled trial that compared the use of surgical and conservative interventions in participants with an ACL rupture.	For adults with acute ACL injuries, we found low-quality evidence that there was no difference between surgical management (ACL reconstruction followed by structured rehabilitation) and conservative treatment (structured rehabilitation only) in patient-reported outcomes of knee function at two and five years after injury.
7	Madelaine et al. (2018) [17]	This study aimed to evaluate the tolerance of conservative management of ACL rupture in children and find predictive factors of ACL reconstruction.	The study was an observational, retrospective study of patients under 18 with MRI-confirmed ACL ruptures from 2007-2017. All patients were under the age of eighteen and were treated conservatively. Two groups were analyzed: operated patients and conservative treatment. The follow-up consultations were scheduled six months after the injury, then every year.	Fifty-three patients were included in this study. The median length of follow-up was 31.5 months [interquartile range (IQR): 22.3-49.3]. The median age at the last follow-up was 14.4 years [IQR: 12.6-15.5]. Nineteen patients (36%) described knee instability at the last follow-up. On the last MRI, 9 (17%) patients had meniscal tears but only one patient (2%) needed a meniscectomy. Twenty-one patients (40%) underwent ACL reconstruction. The 4-year successful conservative treatment rate was 92% (95% CI: 85-98%). Clinical instability at first examination was the only significant predictive factor of bad tolerance of conservative management (p=0.047). The study concluded that conservative management is viable for pediatric ACL ruptures but requires further research on pubertal status impact.
8	Van Yperen et al. (2018) [18]	To compare the long-term treatment outcomes of operative versus nonoperative treatment of ACL ruptures in high-level athletes.	Retrospective pair-matched cohort study, 20-year follow-up of a previous 10-year study Comparison between operative and non-operative treatment of ACL ruptures	There was no significant difference in the rate of knee osteoarthritis between the operative and non-operative treatment groups after 20 years of follow-up. - The rate of meniscectomies performed was also not significantly different between the two groups over the 20-year follow-up period. - There were no significant differences in functional outcomes between the operative and non-operative treatment groups after 20 years.
9	Gföller et al. (2019) [19]	The aim of this study was the evaluation of long-term clinical and radiological outcomes of non-operative treatment of anterior cruciate ligament (ACL) deficiency.	The evaluation was based on objective and subjective scores, instrumented testing, radiographic examination and assessment of sports activity.	Patient satisfaction with conservative treatment of ACL injuries is good despite objective measures indicating increasing degenerative changes.
10	Hoogeslag et al. (2019) [20]	The objective of the study was to evaluate the effectiveness of dynamic augmented anterior cruciate ligament (ACL) suture repair compared to anatomic single-bundle ACL reconstruction in terms of patient self-reported outcomes at 2 years postoperatively.	Randomized controlled trial	It remains inconclusive whether the effectiveness of DA ACLSR is non-inferior to that of ACLR in terms of subjective patient-reported outcomes as measured using the IKDCs. Although DA ACLSR may be a viable treatment option for patients with acute ACL rupture, caution must be exercised when considering this treatment for young, active patients, corresponding to the present study population.
11	Reijman et al. (2021) [21]	To assess whether a clinically relevant difference exists in patients’ perceptions of symptoms, knee function, and ability to participate in sports over two years after rupture of the anterior cruciate ligament (ACL) between two commonly used treatment regimens.	- Open-labeled, multicenter, parallel randomized controlled trial. - Patients aged 18-65 with acute ACL rupture were recruited from 6 hospitals in the Netherlands. - Randomized to early ACL reconstruction or rehabilitation with optional delayed reconstruction. - Primary outcome was IKDC score over 24 months. - Secondary outcomes included other knee scores, return to sport, giving way, and adverse events.	- Patients who underwent early ACL reconstruction had significantly better perceptions of symptoms, knee function, and ability to participate in sports at 2 years compared to those who underwent rehabilitation with optional delayed ACL reconstruction, though the clinical importance is unclear. - Half of the patients randomized to the rehabilitation group did not need surgical reconstruction. - The rehabilitation group had significantly better outcomes in the first 3 months, but the early ACL reconstruction group had better outcomes after 9 months.
12	Park et al. (2021) [22]	To evaluate the result of implementing an initial non-operative treatment program for an acute ACL injury and to find if the timing of initiating the non-operative treatment is significant.	Prospective cohort study	Implementing a non-operative treatment with a brace in the acute phase of ACL injury appears to be an effective and viable option to achieve a reasonable clinical outcome.
13	Van der Graaff et al. (2022) [23]	To investigate why, when, and which patients with an ACL rupture who initially started with rehabilitation therapy required reconstructive surgery.	Study Design: A case-control study (Level 3 evidence) Sample: 82 patients from the COMPARE trial who were initially assigned to rehabilitation therapy plus optional delayed ACL reconstruction	- Most patients (41.5%) who underwent delayed ACL reconstruction received it between 3 to 6 months after starting rehabilitation therapy. - The main reason for delayed ACL reconstruction was instability concerns, reported by 90.2% of patients. - Patients who underwent delayed ACL reconstruction had a significantly younger age and higher preinjury activity level compared to those who did not require surgery.
14	Beard et al. (2022) [24]	To investigate the best management strategy between reconstructive surgery and non-surgical treatment for patients with a non-acute ACL injury and persistent symptoms of instability.	A pragmatic, multicenter, superiority, randomized controlled trial in 29 secondary care National Health Service orthopedic units in the UK	Surgical reconstruction as a management strategy for patients with non-acute ACL injury with persistent symptoms of instability was clinically superior and more cost-effective in comparison with rehabilitation management.

Heterogeneity Analysis

Cochran's Q Test yielded a statistic of 22.53, which measures the degree of heterogeneity among the studies. This value suggests a moderate level of variability in the results. The accompanying p-value for the Q Test is 0.0476, falling below the common threshold of 0.05, which indicates statistically significant heterogeneity among the studies. This finding implies that the observed differences in effect sizes are unlikely to be due to chance alone. Additionally, the I² statistic of 42.31% further reflects moderate heterogeneity, indicating a degree of variability among the studies that is not excessively high. These values are summarized in Table 2.

**Table 2:** Cochran’s Q test: key statistics and p-value

Statistic	Value
Cochran's Q Test	22.53
P-value for Q Test	0.0476
I² Statistic	42.31%

*Publication Bias Assessment* 

**Figure 2:**
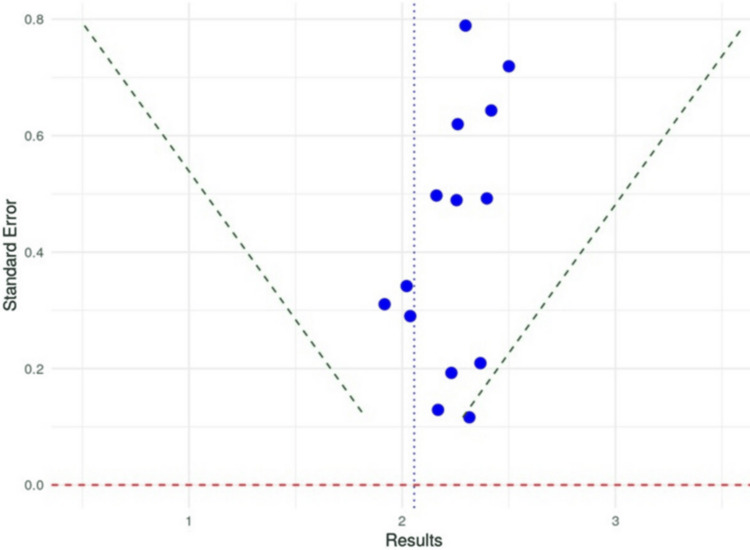
Funnel plot for publication bias

The analysis of effect size as shown in Figure 2, indicates a central value of 2, which serves as an average effect size across various studies. Most data points cluster around this line, suggesting a consistent trend in the reported effects. Studies with lower standard errors, typically larger and more precise, are concentrated toward the bottom of the plot, offering more reliable estimates.

While some studies show effect sizes between 1 and 3, particularly those with higher standard errors, the overall distribution remains balanced around the average effect size of 2. There is a slight tendency for effect sizes to exceed 2, but this is not pronounced, indicating minor heterogeneity. Overall, the funnel plot suggests a strong synthesis of evidence, with little indication of significant publication bias, reinforcing the reliability of the average effect size across the studies.

Further analysis for publication bias was done, using Egger’s test. In the analysis conducted using Egger's test, a weighted regression model was employed, where standard error served as the predictor. This method examines the correlation between effect size and its precision. The t-value calculated was 0.5756, indicating the extent of deviation from the null hypothesis, which assumes that there is no publication bias present.

The analysis included 14 studies, and with 12 degrees of freedom, this reflects the number of independent data points available for estimating variance within the model. This number results from subtracting the two estimated parameters - the intercept and the slope - from the total number of studies.

The p-value obtained was 0.5724, considerably exceeding the typical alpha level of 0.05. This high p-value suggests a lack of statistically significant evidence for funnel plot asymmetry or publication bias among the studies reviewed. Furthermore, the limit estimate (b) was noted at 0.0049, indicating a very minimal positive correlation between effect size and standard error, though this correlation is not of considerable significance.

The CI ranged from -0.2209 to 0.2306 and includes zero, further supporting the conclusion that there is no substantial evidence of bias. This finding implies the possibility that there is no effect at all. Overall, Egger's test did not reveal significant publication bias in the studies analyzed, suggesting that the observed effect sizes are credible and that the asymmetry in the funnel plot does not imply any systematic bias in how results are reported. These are all shown in Table 3.

**Table 3:** Results of weighted regression analysis

Metric	Value
Model	Weighted regression with multiplicative dispersion
Predictor	Standard error
t-value	0.5756
Degrees of Freedom (df)	12
p-value	0.5724
Limit Estimate (as sei → 0)	b = 0.0049
Confidence Interval (CI)	(-0.2209, 0.2306)

Complications and risks associated with conservative management versus surgical intervention in treating ACL injuries: The analysis of complications related to ACL injury treatments revealed an average of approximately 49.83 complications per study, highlighting a significant burden associated with these interventions. The results were statistically significant, with a p-value of less than 0.0001, indicating that the observed complication rates are unlikely to be attributed to chance.

Furthermore, the estimated tau² value of 1,755.33 pointed to considerable heterogeneity among the studies, suggesting that complication rates vary significantly depending on the treatment methods and patient populations involved. The CI for the rate of complications spanned from 25.84 to 73.82, illustrating a wide range of potential outcomes and indicating that the true average complication rate may differ considerably across different studies.

These findings underscore the substantial risk of complications associated with surgical interventions for ACL injuries, which necessitates careful consideration when making treatment decisions. It highlights the importance of individualized patient assessments to balance the risks of surgery against the potential benefits of conservative management options. Lastly, the study calls for further research to refine treatment strategies for ACL injuries, focusing on minimizing complications while achieving positive patient outcomes. These findings are summarized in Table 4.

**Table 4:** Meta-analysis results

Parameter	Value
Number of studies (k)	14
Log-likelihood	-68.1412
Deviance	136.2824
AIC	140.2824
BIC	141.4123
tau² (total heterogeneity)	1755.3252 (SE = 789.0674)
tau (sqrt(tau²))	41.8966
Estimate (mean complications)	49.8294
Standard error (SE)	12.2408
Z-value	4.0708
P-value	<0.0001
Confidence interval (CI)	[25.8378, 73.8210]

**Figure 3:**
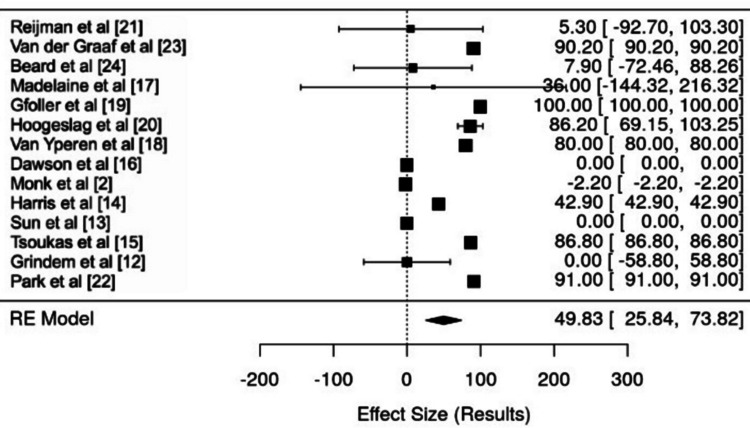
Forest plot of complications associated with ACL treatment Refs. [2,12-24] ACL - anterior cruciate ligament

The forest plot (Figure 3) provides a visual summary of effect sizes from various studies focused on complications associated with ACL treatment. Each study was depicted with a square representing the point estimate and horizontal lines indicating CIs, allowing for an easy comparison of the data.

The effect sizes demonstrated considerable variability, with some studies reporting minimal complications (effect sizes near zero) while others showed more pronounced positive or negative outcomes. This variability was further illustrated by the size of the squares, indicating differences in sample sizes and the precision of estimates across the studies.

Larger squares, like those from studies by Hoogeslag et al. and van Yperen et al., had narrower CIs, signaling higher reliability in their findings [18,20]. In contrast, studies such as Madelaine et al. displayed wider CIs, suggesting less certainty in the results [17].

When assessing statistical significance, the analysis showed that CIs crossing zero indicated non-significant findings in studies like Madelaine et al. and Sun et al. [13,17]. This uncertainty questioned the effectiveness of ACL treatments in those cases. On the other hand, studies with larger effect sizes, like those by Hoogeslag et al. and van Yperen et al., revealed statistically significant outcomes alongside narrow CIs [18,20].

Ultimately, the diamond at the bottom of the forest plot synthesized the overall effect size derived from a random-effects model, estimating it at approximately 49.83 for treatment complications, with a 95% CI of (25.84, 73.82). This indicated a statistically significant positive correlation between ACL treatment and complications, generally implying that the treatments were associated with adverse effects.

Long-term functional outcomes of conservative management compared to surgical intervention in patients with ACL injuries regarding QoL, RTS, and re-injury rates: In assessing reinjury rates, the Conservative group demonstrated a mean rate of 0, highlighting effective management in preventing further injuries. Conversely, the Surgical group reported a mean reinjury rate of 11.1, suggesting that some patients experienced reinjuries following surgery. While these results point to a potential advantage of conservative management in reducing reinjury risk, statistical analysis revealed no significant difference between the two groups.

Regarding QoL, patients in the Conservative group achieved a higher mean score of 64.6 compared to 57.1 in the Surgical group. This indicates that conservative management may contribute to better overall well-being and health satisfaction than surgical intervention.

For RTS, both groups showed an identical mean score of 2, suggesting that the likelihood of resuming athletic activities was comparable for patients in both interventions. This parity implies that while surgical intervention did not enhance RTS outcomes, it was equally effective as conservative management in this respect. The mean reinjury rate, QoL and return to sport are summarized in Table 5.

**Table 5:** Summary of long-term functional outcomes QoL - quality of life, RTS - return to sport

Intervention type	Mean reinjury rate	Mean QoL	Mean RTS
Conservative	0	64.6	2
Surgical	11.1	57.1	2

The T-test results (Table 6) indicated a T-statistic of -1.6587 and a p-value of 0.1483, signifying no statistically significant difference in reinjury rates between the Conservative and Surgical groups. The 95% CI of (-27.55, 5.29) further supports this conclusion, as it includes zero, suggesting that any difference in mean reinjury rates is not statistically significant.

**Table 6:** T-test results

Statistic	Value
T-statistic	-1.6587
Degrees of Freedom (df)	6
P-value	0.1483
95% Confidence Interval	(-27.55, 5.29)

In summary, the findings suggested that conservative management may lead to better QoL outcomes and lower reinjury rates compared to surgical intervention, while both groups demonstrate comparable RTS rates. Nevertheless, the absence of statistically significant differences indicates that further investigation may be necessary to explore these outcomes in larger or more varied populations. This interpretation comprehensively addresses all the variables of interest in evaluating the long-term functional outcomes for ACL injuries.

Discussion

This meta-analysis investigates the comparative effectiveness of conservative management versus surgical interventions for ACL injuries, revealing several important insights into the treatment options available for patients. The analysis produced findings characterized by moderate heterogeneity, as indicated by an I² value of 42.31%. This degree of variability may stem from differences in patient demographics, surgical techniques employed, and protocols for rehabilitation utilized across the various studies reviewed [25].

One of the significant findings from this analysis is that conservative treatment approaches tend to result in lower rates of reinjury compared to surgical interventions, with a p-value of 0.1483, although it is important to note that this result did not reach statistical significance. This observation is consistent with previous studies that suggest conservative management might be a more appropriate option for certain patient populations [2]. Furthermore, the health-related QoL (HRQoL) assessments indicated that individuals receiving conservative management reported higher QoL scores (64.6) compared to those who underwent surgical intervention (57.1). This challenges the longstanding notion that surgical options are inherently superior for all patients [14].

When examining complication rates among the studies analyzed, the mean reported was 49.83 complications per study (CI: 25.84-73.82), which suggests a concerningly high incidence of complications associated with surgical management strategies. This finding emphasizes the necessity for careful patient selection, individualized treatment planning, and risk assessment when considering surgical interventions [7]. Remarkably, the data showed equivalent RTS rates between the conservative and surgical treatment groups (mean RTS = 2), indicating that conservative management may yield similar functional outcomes for patients without complications or specific surgical indications [6].

From a clinical perspective, the implications of these findings are substantial. They prompt a reevaluation of the traditional paradigm that positions surgical treatment as the default or preferred course of action for all ACL injuries. Instead, the results advocate for a more personalized approach to treatment that considers individual patient characteristics, lifestyle factors, and specific treatment goals [21]. Additionally, the results stress the importance of ongoing long-term follow-up to accurately assess the efficacy of treatment strategies over time.

However, it is essential to acknowledge certain limitations inherent in this study. The moderate heterogeneity observed among the studies suggests that the findings may not be universally applicable to all patient populations and scenarios. Furthermore, the variability in follow-up duration and the different outcome measures utilized across studies could significantly impact the generalizability of the conclusions drawn from this meta-analysis. Overall, while the results provide valuable insights into ACL treatment options, they also highlight the need for future research to address these limitations and further clarify the factors influencing treatment outcomes.

## Conclusions

In conclusion, conservative management may provide similar benefits for selected ACL injuries regarding functional outcomes, reinjury rates, and QoL. These results contribute to the growing evidence supporting a more cautious and individualized treatment approach for ACL injuries, where management is tailored to each patient's specific needs rather than relying on a one-size-fits-all protocol.

Further research is needed to identify the specific patient cohorts that are most likely to benefit from each treatment strategy and to standardize outcome measures, which will facilitate comparisons among different studies. These conclusions hold significant implications for clinical practice. They suggest that clinicians should consider conservative management as a suitable first-line treatment for select patients, rather than automatically opting for surgical intervention. Ultimately, these findings may inform future treatment approaches following ACL injuries, promoting a more cost-effective and patient-centered strategy.
